# The Role of Sex in Genetic Association Studies of Depression

**DOI:** 10.20900/jpbs.20220013

**Published:** 2022-12-30

**Authors:** Karin F. Hoth, Kirsten Voorhies, Ann Chen Wu, Christoph Lange, James B. Potash, Sharon M. Lutz

**Affiliations:** 1. Department of Psychiatry and Iowa Neuroscience Institute, University of Iowa, Iowa City, IA 52242, USA; 2. Precision Medicine Translational Research Center (PROMoTeR), Department of Population Medicine, Harvard Pilgrim Health Care Institute and Harvard Medical School, Boston, MA 02215, USA; 3. Department of Biostatistics, Harvard T.H. Chan School of Public Health, Boston, MA 02115, USA; 4. Department Psychiatry and Behavioral Sciences, Johns Hopkins University School of Medicine, MD 21287, USA

**Keywords:** depression, genome wide association studies, sex stratified, SNP by sex interaction

## Abstract

Depression is the most common mental illness in the U.S. affecting nearly 40 million adults age 18 years and older. Depression has both genetic and environmental influences. In addition, women are more likely to be affected by depression than men. However, the relationship between genes and depression is complex and may be influenced by sex. Understanding the genetic basis of sex-specific differences for depression has the potential to lead to new biological understanding of the etiology of depression in females compared to males and to promote the development of novel and more effective pharmacotherapies. This review examines the role of sex in genetic associations with depression for both genome-wide association and candidate gene studies. While the genetic association signals of depression differ by sex, the role of sex in the heritability of depression is complex and warrants further investigation.

## INTRODUCTION

Depression has a major impact on health and significant financial implications. Depression is common and affects over 300 million people worldwide [1]. At least one in five US adults experience depression in their lifetime [2]. According to the World Health Organization (WHO), depression is ranked as the single largest contributor to global disability and close to 800,000 people die from suicide annually [3]. Depression is a complex condition with both environmental and genetic risk factors that may vary by sex.

Women have nearly twice the risk of developing depression as men [4–8]. Certain subtypes of depression are particularly more common in women [9,10], such as seasonal depression [11]. Furthermore, past research suggests that women with depression tend to have different symptom profiles than men and experience a more significant impact from depression on daily functioning [12–15]. Women also respond differently to pharmacological interventions [16–21]. Sex-differences in antidepressant efficacy have been attributed to sex-based physiological differences, differences in behavior, and conditions like pregnancy and menopause [16].

In addition to sex differences, there is substantial evidence that depression is heritable [22] and a polygenic disorder (due to the combined effects of many genetic variants) [23]. The heritability of depression is estimated to be approximately 40% based on twin studies [22]. Despite the strong evidence for the heritability of depression, early efforts to identify specific genetic variants associated with depression using genome-wide association studies (GWAS) had limited success as they were underpowered [24,25]. The last several years have been marked by rapid progress in the field given advances in statistical methods and increasing sample sizes, especially with the UK Biobank [19,20,26–29]. In recent GWAS, a large number of single nucleotide polymorphisms (SNPs) have been associated with several depression phenotypes [20,21]. Patterns of SNP associations have been shown to differ across nine symptoms of depression [30]. These results provide evidence that current depressive symptoms are genetically heterogeneous [30] and sex differences in the genetic architecture of depression have been observed [31]. However, the role of sex in genetic association studies of depression is unclear [32]. In this review, we explore the role of sex in GWAS and candidate gene studies of depression.

## GWAS OF DEPRESSION AND SEX

Despite the known sex differences in the epidemiology of depression, only a few GWAS of depression have stratified by sex as summarized in Table 1. First, a GWAS meta-analysis of depression in Generation Scotland and UK Biobank found one SNP on chromosome 3p22.3 (*CRTAP*) that was significantly associated with major depressive disorder (MDD) among males, but not among females or participants considered together [33]. Second, among 12,310 adults in the Hispanic Community Health Study/Study of Latinos (HCHS/SOL), no SNPs were genome-wide significant in the overall GWAS for probable depression. However, in the sex-stratified analyses, seven genome-wide significant SNPs were identified: rs72662446 (*LOC107986945*) among females and rs111365740 (*ANXA5*), rs9709324 (*METRNL*), rs113403132 (*ZNF285*), rs6993028, rs144850488 (*ZNF285*), rs17800303 (*ZNF285*) among males [34]. Third, using whole exome sequencing data from 1000 Korean patients with depressive disorder, three genes were associated with increased risk among females (*PDE4A*, *FDX1L, MYO15B*) using a significance threshold of 5 × 10^−5^ [31]. Finally, a recent large GWAS found separate SNPs associated with MDD for men and women in the Netherlands Twin Register (NTR) and Netherlands Study of Depression and Anxiety (NESDA); 40 male-specific and 56 female-specific MDD-associated SNPs were identified using a significance threshold of 1 × 10^−4^ [35]. Using a threshold of 1× 10^−5^, one SNP [*LGSN*] was associated with MDD among males and 6 SNPs in 4 genes [*PCLO*, *LAPTM4A/SDC1,C18orf62, SPC24/KANK2*] were associated with MDD among females. These studies demonstrate that SNPs may be associated with depression among males or females, but would not be found in the overall GWAS unless sex stratified analyses were considered. However, these studies did not formally test if there was a statistically significant sex difference for the genetic associations of depression.

In addition to stratifying by sex, a few studies have examined genome-wide SNP-by-sex interactions on depression. The GWAS for the Netherlands NESDA and NTR populations stratified by sex and also found 1 SNP-by-sex interaction using a significance level of *p* < 10^−5^ and 38 SNP-by-sex interactions using a significance level of *p* < 10^−4^ [35]. In the PGC (Psychiatric Genomics Consortium) and iPsych consortiums, among 32,408 MDD cases and 109,946 controls using a significance threshold of 5 × 10^−7^, a genome-wide SNP-by-sex interaction study found two significant interactions for MDD on chromosome 1p12 and 17q21 [*SPAG17, ZNF385C*] and a significant interaction for recurrent MDD on chromosome 1p12 [*SPAG17*] [36].

## CANDIDATE GENE STUDIES OF DEPRESSION AND SEX

The majority of candidate gene studies for depression and sex have focused on the serotonin transporter gene (*5-HTT*) on chromosome 17q11, particularly polymorphisms of the promoter region of the gene as summarized in Table 2. There is evidence that variation in the 5-HTT promotor region interacts with stress in the presentation of depression, and this has been examined in some studies via 3- and 4- way interactions with sex. Among a cohort of 17–18 year old students (*N* = 1482), there was a significant association between the promoter length polymorphism 5HTTLPR (chromosome 17q11, gene *SLC6A4*) and depression among females, but not in the cohort as a whole [37]. Among 252 Chinese adolescents, there was not a significant association between 5-HTTLPR and depression in the entire cohort, but there was a significant 5-HTTLPR-by-stress interaction on depression among females only [38]. In the National Longitudinal Study of Adolescent Health (Add Health), there was a significant interaction of 5-HTTLPR and family support on the development of depression among males and a marginally significant interaction among females [39]. Another study in Add Health found an interaction of 5-HTTLPR and perceived stress on depressive symptoms among females, but not males [40]. In patients with pain, a 5HTR2A promoter variation (rs6311) was found to have sex-dependent modulatory effects on depression and physical function [41]. In addition, a study found a significant three-way interaction among 5-HTTLPR, sex, and environmental stress (i.e., being a caregiver and lower childhood SES) on depression [42]. Among a cohort of adolescents, a significant four-way interaction of 5-HTTLPR, the monoamine oxidase A-upstream variable number tandem repeat (MAOA-uVNTR), negative life events at age 13, and sex was observed for depressive symptoms at age 15 [43]. In a study of 16–19 year old school students in Sweden, the short 5-HTTLPR allele was associated with the development of depressive symptoms in response to environmental stressors for females but not males [44]. A study of 10–12 year old children found that increased depressive symptoms were associated with 5-HTTLPR only for females who experienced childhood emotional abuse [45]. A previously published review and methodological analysis examined the inconsistencies between 15 studies that found significant gene-by-environment interactions between 5-HTTLPR and environmental adversity on the onset of depression, with two studies that reported negative findings [46]. In several of these studies, there was not an overall association between the variant and depression, but there was a significant association among females.

Additional candidate gene studies have examined differences in the association between polymorphisms in other regions and depression by sex. Among 2500 individuals in Add Health, dopamine receptor variants in *DRD2* and *DRD4* were associated with depressive symptoms among male adolescents and young adults [47]. For candidate genes of monoamine neurotransmission (*SLC6A4*, *TPH2, COMT, MAOA ,DRD1-DRD5*), among 5225 participants from the Northern Finland Birth Cohort 1966, there was a significant SNP-by-sex interaction on depression for a variant on chromosome 11q23 (*DRD2*) [48]. For the EH-domain containing 3 (*EHD3*) gene on chromosome 2p23.1, which encodes a protein participating in endosome protein trafficking, two SNPs (rs619002, rs644926) were associated with MDD among females [49]. Among patients referred for cardiovascular evaluation (*N* = 1368), a significant interaction was found for a haplotype in the *LTA4H* gene and sex on depression pointing to a potential sex-specific role for leukotriene metabolism in depression [50]. Among Chinese Han adolescents after the 2008 Wenchuan earthquake, there was evidence of a longitudinal association with depression between the Val66Met SNP in the brain derived neurotrophic factor (*BDNF*) gene and sex [51]. An additional study found a three-way interaction between the *Val66Met* SNP, unsupportive relationships and sex for depressive symptoms [52]. The association between the gene *CRHR1* and MDD has been shown to differ by sex in separate analyses of Brazilian adults [53]. Variants in the *CRHR1* gene have also been shown to have a protective effect on developing depression after childhood trauma among males, but not females [54]. There was a significant association between disheveled 3 (*DVL3*) and glycogen synthase kinase 3 beta (*GSK3β*) variants, both involved in the Wnt pathway that is implicated in oxidative stress, and the risk of developing MDD among Chinese Han women [55]. An additional study found a significant sex-by-SNP interaction on depression for *APOE* in patients with Alzheimer’s disease such that women with the *APOE* epsilon4 allele were almost 4 times more likely to have depression than those without the *APOE* epsilon4 genotype [56]. In a sex-stratified analysis, there was a significant association between a variation in the *HTR2C* gene (rs6318 (Ser23Cys)) and depression among females, but not males [57]. rs6318 (Ser23Cys) has previously been associated with cortisol release and short-term changes in affect in response to stress in the lab [58]. These studies show the same trend as observed above: the association of the SNPs with depression may be observed in the sex-stratified analysis, but not in the overall sample.

## DISCUSSION

The current review describes previously published studies examining the role of sex in genetic associations with depression. Table 1 presents previous GWAS and Table 2 presents candidate gene studies of depression that have been stratified by sex or examined SNP by sex interactions. As seen in the tables, most associations reported in the literature did not replicate in independent studies. None of the candidate SNPs that were identified as significant in Table 2 had a *p*-value less than 1 × 10^−5^ for the sex-stratified GWAS in the Hispanic Community Health Study/Study of Latinos (HCHS/SOL) [34]. A previous study found that poor replication of candidate genes for MDD using GWAS data may point to publication bias, false-positive findings in previous candidate gene studies, or heterogeneity of the MDD phenotype [66]. These same issues may lead to poor replication for candidate genes for sex differences in depression. In addition, the lack of replication could be due to several other factors including: low sample size and environmental factors. It is also possible that the sex differences in depression are due more to epigenetics than genetics.

Additionally, while the majority of studies examining sex differences in genetic associations of depression are candidate gene studies, GWAS may provide a more promising avenue for the discovery of sex-specific effects. Candidate gene studies have less of a multiple testing burden than GWAS. While the significance threshold for GWAS is usually 5 × 10^−8^, the threshold for candidate gene studies can vary, using a significance threshold of 0.05 or a Bonferroni correction of 0.05 divided by the number of SNPs or regions tested. However, candidate gene studies of depression may be underpowered to detect true positive gene-based associations and robust sex-specific effects. For example, the evidence implicating serotonin transporter genes in depression is weak and inconsistent. More GWAS examining sex differences in genetic associations of depression are needed.

In conclusion, there is a complex relationship between depression, heritability, environment, and sex. The heritability of depression has been shown to potentially differ by sex as genetic associations with depression have been observed in sex-stratified analyses that were not observed in the overall cohort. However, why women experience depression at twice the rate of men remains unknown, though reproductive hormones are thought to be one important factor. As stated in the current National Institute of Mental Health (NIMH) Strategic Plan for Research, investigations of sex differences related to mental health are needed to provide information essential to develop precision medicine and personalized interventions [67]. Understanding the genetic basis of sex-specific differences in depression has the potential to lead to new biological understanding of the etiology of depression in women vs. men, and to promote the development of novel and more effective pharmacotherapies and other interventions. For example, in 2019 the FDA approved the first sex-specific treatment, brexanolone, for postpartum depression. Other sex-specific treatments await discovery. Further analyses with improved statistical methods to detect genetic risk variants for depression that vary by sex have the potential to identify new pathways in which novel targets can be tested for their therapeutic potential and better personalized timepoints for intervention can be identified.

## Figures and Tables

**Table 1.** Summary of results for the GWAS of depression and sex studies. Study ref. refers to the reference number of the corresponding manuscript. The replication column indicates whether the study tested for replication, failed to replicate, or replicated the association.

Chr	Gene/nearest gene	SNP	Allele	Freq	Sample size	Study Ref.	Outcome	Method	OR (SE) or *β* (SE)	*p-value*	Assoc. in males or females	Sign. Threshold	Replicated
1p12	*SPAG17*	rs9428240	T/C	0.41	36,078	[36]	MDD	Interaction	*β*: −0.18 (0.04)	1.6 × 10^−7^	M: *β* = 0.09 (0.03), *p* = 8.4 × 10^−4^; F: *β* = −0.09 (0.02), *p* = 6.4 × 10^−5^	5 × 10^−7^	No
1p12	*SPAG17*	rs61138090	-	0.41	23,661	[36]	Recurrent MDD	Interaction	*β*: −0.24 (0.05)	1.4 × 10^−7^	M: *β* = 0.14 (0.04), *p* = 2.1 × 10^−4^; F: *β* = −0.11 (0.03), *p* = 1.0× 10^−4^	5 × 10^−7^	No
2p24	*LAPTM4A*/*SDC1*	rs12471796	A/G	0.30	2206	[35]	MDD	Stratified	OR: 1.37	3.3× 10^−6^	Females	1 × 10^−5^	No
2p24	*LAPTM4A*/*SDC1*	rs7565124	A/G	0.30	2206	[35]	MDD	Stratified	OR: 1.38	3.1 × 10^−6^	Females	1× 10^−5^	No
2q24	-	rs12692709	T/C		3356	[35]	MDD	Interaction	-	5.8 × 10^−6^	M: *β* = 0.09 (0.03), *p* = 8.4× 10^−4^; F: *β* = −0.09 (0.02), *p* = 6.4× 10^−5^	1 × 10^−5^	No
3p22	CRTAP	rs4478037	A/G	0.08	43,062	[33]	MDD	Stratified	*β*: 0.29 (0.05)	2.3 × 10^−8^	Males	5 × 10^−8^	No
4q27	ANXA5	rs111365740	C/G	0.03	5046	[34]	Depressive symptom score*	Stratified	*β*: −1.86 (0.34)	3.7 × 10^−8^	Males	5 × 10^−8^	Failed to replicate
6q12	*LGSN*	rs9352774	C/A	0.11	1150	[35]	MDD	Stratified	OR: 1.93	2.3 × 10^−6^	Males	1 × 10^−5^	No
7q21	*PCLO*	rs2715148	A/C	0.48	2206	[35]	MDD	Stratified	OR: 0.74	5.6 × 10^−7^	Females	1 × 10^−5^	No
7q21	*PCLO*	rs2107828	A/T	0.46	2206	[35]	MDD	Stratified	OR: 0.75	2.3 × 10^−6^	Females	1 × 10^−5^	No
8p21	-	rs6993028	G/A	0.02	4832	[34]	Depressive symptom score**	Stratified	*β*: −2.01 (0.35)	1.3 × 10^−8^	Males	5 × 10^−8^	Failed to replicate
8q12	LOC1079 86945/FAM11OB	rs72662446	C/T	0.03	7263	[34]	Depressive symptom score*	Stratified	*β*: −1.85 (0.32)	6.4 × 10^−9^	Females	5 × 10^−8^	Failed to replicate
17q21	*ZNF385C*	rs147515485	T/C	0.02	66,534	[36]	MDD	Interaction	*β*: −0.47 (0.09)	4.6 × 10^−7^	M: *β* = 0.30 (0.07), *p* = 4.4 × 10^−5^; F: *β* = −0.19 (0.06), *p* = 1.6 × 10^−^3	5 × 10^−7^	No
17q25	*MYO15B*	rs820182	-	0.11	1000	[31]	MDD (only depressive subjects)	Difference in allele freq. by sex	OR: 1.9	1.5 × 10^−5^	-	5 × 10^−5^	No
17q25	*METRNL*	rs9709324	C/T	0.03	5046	[34]	Depressive symptom score*	Stratified	*β*: −2.08 (0.38)	4.2 × 10^−8^	Males	5 × 10^−8^	Failed to replicate
18q23	*c18orf62*	rs2584466	C/T	0.46	2206	[35]	MDD	Stratified	OR: 0.76	8.9 × 10^−6^	Females	1 × 10^−5^	No
19q13	ZNF285/ZFP112	rs144850488	G/A	0.05	4832	[34]	Depressive symptom score**	Stratified	*β*: −1.43 (0.26)	2.7 × 10^−8^	Males	5 × 10^−8^	Failed to replicate
19q13	ZNF285/ZFP112	rs17800303	C/A	0.05	4832	[34]	Depressive symptom score**	Stratified	*β*: −1.35 (0.24)	3.6 × 10^−8^	Males	5 × 10^−8^	Failed to replicate
19q13	ZNF285	rs113403132	G/A	0.04	4832	[34]	Depressive symptom score**	Stratified	*β*: −1.45 (0.25)	7.7 × 10^−9^	Males	5 × 10^−8^	Failed to replicate
19p13	*SPC24*	rs7251031	G/T	0.32	2206	[35]	MDD	Stratified	OR: 0.75	9.8 × 10^−6^	Females	1 × 10^−5^	No
19p13	PDE4A	rs201432982	-	0.05	1000	[31]	MDD (only depressive subjects)	Difference in allele freq. by sex	OR: 2.6	2.9 × 10^−5^	-	5 × 10^−5^	No
19p13	FDX1L/RAVER1	rs62640397	-	0.05	1000	[31]	MDD (only depressive subjects)	Difference in allele freq. by sex	OR: 2.3	3.7 × 10^−5^	-	5 × 10^−5^	No

- = the information was not available in the paper; Depressive symptom score

* = Depressive symptom score, after adjusting for medication use; Depressive symptom score

** = Depressive symptom score (excl. medication users); For the association in males or females,

M = males, F = females.

**Table 2.** Summary of results for the candidate gene studies of depression and sex. Study ref. refers to the reference number of the corresponding manuscript.

Chr.	Gene/nearest gene	SNP	Sample size	Study Ref.	Outcome	Method	OR (SE) or *β* (SE)	*p-value*	Assoc. in males or females	Sign. Threshold	Replication
2p23	EHD3	rs619002	531	[49]	MDD	Stratified	OR: 0.47	4 × 10^−3^	Female	0.04	No
2p23	EHD3	rs644926	531	[49]	MDD	Stratified	OR: 0.49	7 × 10^−3^	Female	0.04	No
3q13	GSK3β	rs334558	751	[55]	MDD	Stratified	-	<0.01	Female	0.05	Yes [59]
3q13	GSK3β	rs6438552	751	[55]	MDD	Stratified	-	<0.01	Female	0.05	Age of onset bipolar I disorder among females. [60]
3q27	DVL3	rs1709642	751	[55]	MDD	Stratified	-	<0.01	Female	0.05	Yes, rs1969253 and MDD in females [23]
11p14	BDNF	Val66Met	705	[51]	Depression	Interaction: SNP × sex	OR: 1.77, 2.69, 2.22 (6, 12, 18 mos.)	3 × 10^−3^; <1 × 10^−3^; 1 × 10^−3^	All	0.05	No
11p14	BDNF	Val66Met	945	[52]	Depressive scores	Interaction: SNP × sex × unsupportive relationships	*β*: 5.08 (1.82)	5 × 10^−3^	All	Bonf.	Yes [61,62]
11p15	DRD4	-	1,095	[47]	Depressive symptoms	Stratified	*β*: 0.24	0.01	Male	0.05	No
11q23	DRD2	-	1,095	[47]	Depressive symptoms	Stratified	*β*: 0.06	7 × 10^−3^	Male	0.05	No
11q23	DRD2	rs4274224	5,225	[48]	Depression	Interaction: SNP × sex		0.02	M: *β*/*p* 0.02/6 × 10^−4^	0.05	No
12q23	LTA4H	Haplotype	1,368	[50]	Depression	Interaction: Haplotype × sex	-	0.03	All	Perm*	No
12q23	LTA4H	Haplotype	416	[50]	Depression	Stratified	OR: 0.28	8 × 10^−3^	Female	Perm*	No
13q14	HTR2A	rs6311	224	[41]	Depression	Interaction: SNP × sex × phys. function	-	5 × 10^−3^	All	0.025	No
17q11	SLC6A4	5HTTLPR	717	[37]	Depression	Stratified	OR: 0.42	0.03	Female	-	No
17q11	SLC6A4	5HTTLPR	131	[38]	Depressive symptoms	Interaction w. stress; sex stratified	*β*: 0.08 (0.02)	<1 × 10^−4^	Female	0.05	No
17q11	SLC6A4	5HTTLPR	572	[39]	Depression	Interaction w. family support; sex stratified	*β*: 0.31 (0.09)	< 0.01	Male	0.05	No
17q11	SLC6A4	5HTTLPR	458	[39]	Depression	Interaction w. family support; sex stratified	*β*: −0.09 (0.05)	0.08	Female	0.05	No
17q11	SLC6A4	5HTTLPR	925	[40]	Depression	Interaction w. perceived stress; sex stratified	*β*: 0.18 (0.09)	2 × 10^−3^	Female	0.05	No
17q11	SLC6A4	5HTTLPR	288	[42]	Depression scores	Interaction w. sex and environment	-	<3 × 10^−3^	All	0.05	Yes, 142 subjects [42]
17q11	SLC6A4	5HTTLPR	309	[43]	Depressive symptoms	Interaction w. sex, MAOA, negative life events	*β*: −1.450 (0.555)	9 × 10^−3^	All	-	Yes, in females [63]
17q11	SLC6A4	5HTTLPR	81	[44	Depressive symptoms	Interaction w. residence type; sex stratified	-	2 × 10^−3^	Male	-	No
17q11	SLC6A4	5HTTLPR	81	[44]	Depressive symptoms	Interaction w. separated families; sex stratified	-	2 × 10^−3^	Male	-	No
17q11	SLC6A4	5HTTLPR	119	[44]	Depressive symptoms	Interaction w. traumatic conflicts; sex stratified	-	2 × 10^−3^	Female	-	No
17q11	SLC6A4	5HTTLPR	200	[44]	Depressive symptoms	Interaction w. sex		3 × 10^−4^	All	-	No
17q11	SLC6A4	5HTTLPR	98	[45]	Depressive symptoms	Interaction w. childhood abuse; sex stratified	*β*: 0.31	0.03	Female	0.05	No
17q21	CRHR1	rs110402	310	[53]	MDD	Stratified	OR: 0.70	0.04	Female	0.05	
17q21	CRHR1	rs878886	314	[53]	MDD	Stratified	OR: 1.68	0.02	Male	0.05	Panic disorder [64] and fear acquisition [65]
17q21	CRHR1	rs110402	1059	[54]	BDI scores	Interaction w. childhood abuse; sex stratified	-	0.03	Male	0.05	No
19q13	APOE	-	323	[56]	Depression	Sex interaction	OR: 3.83	0.05	All	0.05	No
Xq23	HTR2C	rs6318	2712	[57]	Depressive symptoms	Interaction w. life stress; sex stratified		0.02	Female	0.05	No
Xq23	HTR2C	rs6318	41	[58]	Depressive mood	Interaction w. laboratory stress; sex stratified		6 × 10^−3^	Male	0.05	No

For the significance threshold column, perm

* = Empirical sig. levels determined by permutation tests,

Bonf. = Bonferroni correction.

## Data Availability

Data sharing not applicable to this article as no datasets were generated or analyzed during the current study.

## ABBREVIATIONS

Add Health: National Longitudinal Study of Adolescent Health
GWA: genome-wide association
MDD: major depressive disorder
SNPs: single nucleotide polymorphisms
